# sEMG: A Window Into Muscle Work, but Not Easy to Teach and Delicate to Practice—A Perspective on the Difficult Path to a Clinical Tool

**DOI:** 10.3389/fneur.2020.588451

**Published:** 2021-02-05

**Authors:** Bernard J. Martin, Yadrianna Acosta-Sojo

**Affiliations:** SensoriMotor Systems-and Human Performance Laboratory, Center for Ergonomics, Department of Industrial and Operations Engineering, University of Michigan, Ann Arbor, MI, United States

**Keywords:** surface electromyography, education, clinical application, sensorimotor asymmetries, sensorimotor system gain, force control, hand dominance

## Abstract

Surface electromyography (sEMG) may not be a simple 1,2,3 (muscle, electrodes, signal)-step operation. Lists of sEMG characteristics and applications have been extensively published. All point out the noise mimicking perniciousness of the sEMG signal. This has resulted in ever more complex manipulations to interpret muscle functioning and sometimes gobbledygook. Hence, as for all delicate but powerful tools, sEMG presents challenges in terms of precision, knowledge, and training. The theory is usually reviewed in courses concerning sensorimotor systems, motor control, biomechanics, ergonomics, etc., but application requires creativity, training, and practice. Software has been developed to navigate the essence extraction (step 4); however, each software requires some parametrization, which returns back to the theory of sEMG and signal processing. Students majoring in Ergonomics or Biomedical Engineering briefly learn about the sEMG method but may not necessarily receive extensive training in the laboratory. Ergonomics applications range from a simple estimation of the muscle load to understanding the sense of effort and sensorimotor asymmetries. In other words, it requires time and the basics of multiple disciplines to acquire the necessary knowledge and skills to perform these studies. As an example, sEMG measurements of left/right limb asymmetries in muscle responses to vibration-induced activity of proprioceptive receptors, which vary with gender, provide insight into the functioning of sensorimotor systems. Beyond its potential clinical benefits, this example also shows that lack of testing time and lack of practitioner's sufficient knowledge are barriers to the utilization of sEMG as a clinical tool.

## sEMG Challenges and Illustrative Examples

It is acknowledged that Neuroscience started with Ambroise Paré (1510–1590), who is credited for systematic “empirical observation…, and methodology for evidence-based medicine” (1, 2). Collecting observations and evidence became easier with the development of instrumentation, allowing the exploitation of physiological signals, as evidenced first by the invention of the stethoscope by Laënnec (3), for exploiting cardio-pulmonary sounds. According to a summary of EMG history by Raez et al. (4), Luigi Galvani demonstrated in 1792 that muscle contraction could be initiated by electrical stimulation. However, it was not until four decades after the invention of the stethoscope that Emil Du Bois-Raymon (1849), founder of electrophysiology (5), showed it was possible to record the electrical activity generated by muscle contraction. This was termed “electromyography” by Marey in 1890 (6), now shortened to EMG, and in particular surface EMG (sEMG) when collected by a non-invasive technique. Since then, electrophysiological signals have been submitted to the torture of various mathematical tools to confess to “romantic lies and *novelistic* truth” [to borrow from Girard (7)]. In other words, the signals create the desire to seize upon expected cryptic information emanating from the object/system of interest. Nevertheless, mimetic evolutions of processing techniques have revealed a wealth of information. In the case of the sEMG, which is the focus here, useful information ranges from force exertion, or muscle load, to the recruitment/control of motor units and disorders effects and is extensively exploited in research (see this editorial project).

The muscle, source of EMG signals, is under the microscope of many fields of Neuroscience, and an allusion to all perspectives is beyond the scope of the present work, which is limited to EMG applications in Ergonomics and Occupational Biomechanics. These fields/disciplines are generally included in engineering, health and safety, and kinesiology schools/departments in the United States and Canada. Within these fields, there are three main categories of application. The first is to use EMG to describe the magnitude and pattern of muscle activity, also called *muscle load or muscle recruitment*. For example, during a task involving overhead reaches/manipulations there may be interest in knowing which of several shoulder muscles are involved, at what time, and with what intensity level [e.g., (8–10)]. The second application is using EMG to predict the forces generated by one or more muscles. Such predictions can be of use in detailed task analyses or for evaluating forces predicted using a biomechanical model (e.g., 3D SSPP™, University of Michigan). The third main application of EMG is to estimate the presence or extent of localized muscle fatigue [see for review (11–13)]. Return-to-work assessment is also receiving consideration; however, current utilization and information on this application are rather limited [(14); and papers in this editorial project].

The following comments express the perspective of lecturers and lab instructors, primarily in engineering but also in other fields. Their opinions are mostly derived from experience and exchanges between investigators as published data on the topic are not available, as far as we know. Two major types of hurdles, which are not specific to these fields, constrain the teaching of EMG as an ergonomic assessment and investigative tool. First, a number of methods designed to evaluate the risk factors associated with work-related musculoskeletal disorders [see for review (12, 15)] must be reviewed and presented, which requires several lectures within a course. The time-dependent risk factors to be identified and quantified include posture, force exertion, repetition, contact stress, vibration, and temperature. Hence, to complement or replace time-consuming observations, most methods rely on “sensors” (now mostly wireless wearable sensors), corresponding signals, and processing techniques designed to quantify the severity indicators for each risk factor. Thus, in addition to the EMG, a broad range of exposure assessment techniques (e.g., biomechanical modeling, upper limbs, and whole-body observation-based methods such as OWAS, OCRA, RULA, NQ, and NLG [see (12), force platforms, video and sensor-based motion analysis, vibration measurement, etc.]) has to be covered with greater or lesser emphasis as a function of each factor's prevalence in occupational activities. The three categories of EMG applications mentioned above (pattern, force/muscle load, fatigue) confer a high significance to the EMG as it relates to the engine powering all “activities.” However, in engineering school graduate programs, in which ergonomics and biomechanics are taught, the whole time dedicated in one course to the EMG is on average 1.5 lectures or about 2 h. The second constraint includes the characterization of the EMG itself and its associated interpretation, as today sensor technology is no longer an issue with “cleaner” signals (thanks to miniaturization, wireless signal transmission, material science, and electronics).

**Figure 1:**
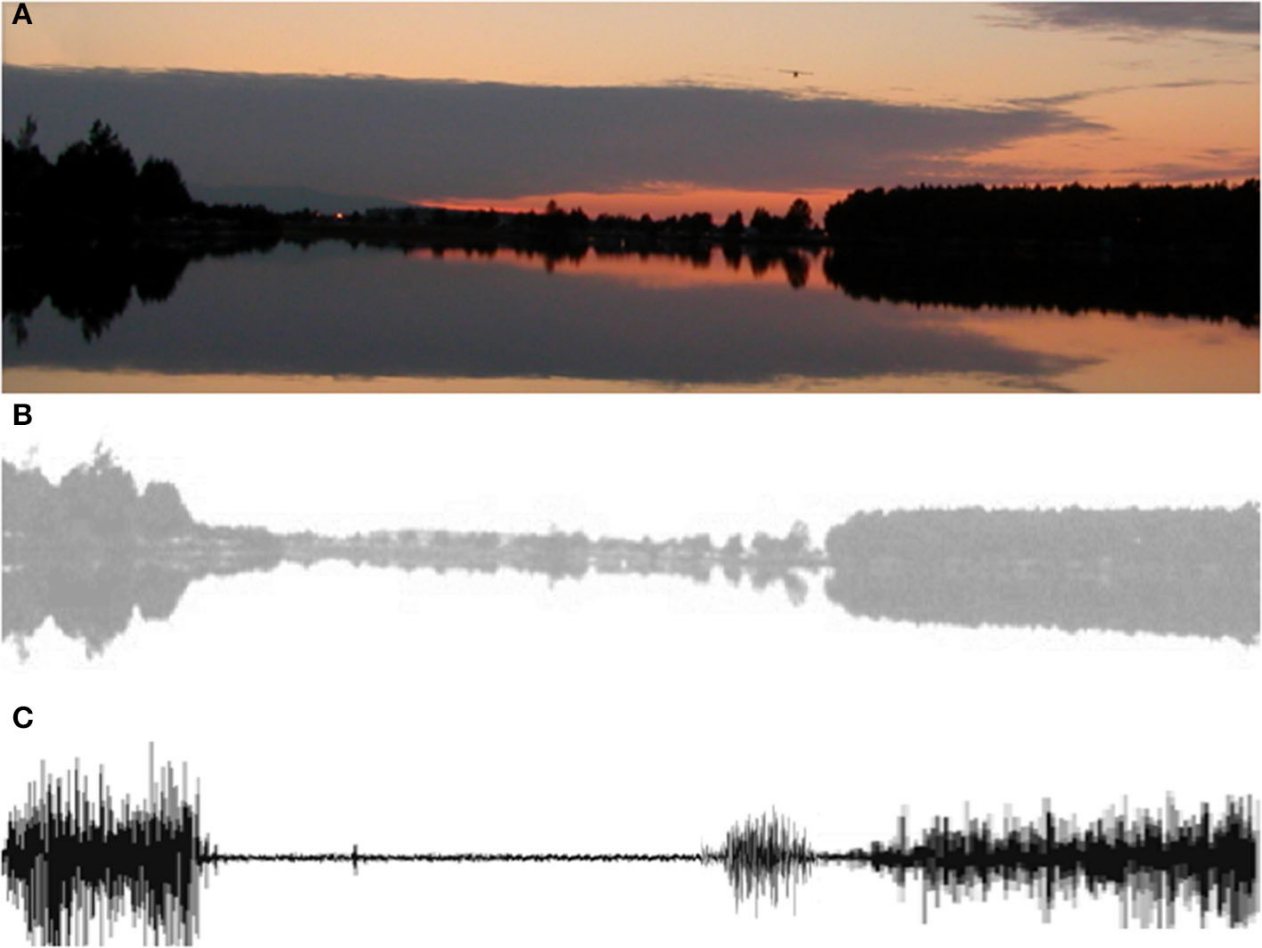
The EMG forest or forest EMG. After adjusting the color **(B)**, the tree line, and its reflection over the dark water of the lake (unknown photographer) at sunset **(A)** produce an EMG-like signal **(C)**. Note that counting/identifying trees in the forest profile can be achieved by using filtering and different visual perspectives of the same scene—as done by the processing of photos obtained with multiple cameras in cutting-edge smart phones. Applied to the EMG signal for motor unit decomposition, a similar process is achieved using multiple comparisons of signals recorded by high-density electrode arrays (see text).

Like many electrophysiological signals, such as the EEG, the EMG displays a noise-like complexion, which blurs the lines between truth and fiction. For example, the profile of a forest mirrored in a dark lake can morph into an EMG by discoloration (Figures 1A,B) and resemble a real EMG (Figure 1C). Hence, extracting meaningful information from that signal becomes a delicate exercise, including also detection and usage of the electromyography (EMG) discipline (16). Among these three components and their multiple issues starting from the anatomy of the muscle to the isolation of motor unit activity and the locus of muscle contraction [e.g., (13)], a number of topics cannot be presented in an easy-to-follow user manual. Although adequate recommendations concerning the placement of electrodes by SENIAM [see (17)] and the reporting of EMG data and measurement systems (www.isek.org) (13, 18) have been published, hands-on experience cannot be replaced by textbooks, lectures, or class demonstrations but must be based on previous theoretical knowledge. The following points summarize the main areas of difficulty for the novice.

- Electrode Usage. The vast majority of currently used electrodes are bipolar. However, the utilization of recently developed electrodes arrays contributes to some complexity that needs to be mastered, leading to significant benefits [(13); and this editrorial project].
- Electrode Placement. This operation, dependent on anatomy and muscle structure, and EMG susceptibility to many factors, is only mastered by hands-on experience. It takes experience to observe cross talk, impedance, artifacts, innervation zone, and electrode migration with skin movements, to cite only a few phenomena. Hence, getting a reliable signal requires the trainer to teach attention to detail and insistence on visual inspection of the raw signal, which can be elusive considering the noise mimetic façade.
- Signal acquisition and processing. Resolution, sampling frequency, gain and saturation, signal-to-noise ratio, non-linearity, sampling window, and calibration are notions rarely familiar to students in our discipline. In addition, being a combination of muscle action potentials, decoding the temporal and spatial summations forming the sEMG is facilitated by the use of high-density electrode arrays and requires the utilization of simple to complex algorithms to characterize the muscle contraction status [e.g., see free tutorials by (19, 20)] and its eventual consequences, such as fatigue [e.g., (21–23)]. Hence, filtering and smoothing and frequency analysis are primary notions to assimilate. All these features require a course in signal processing to bridge the frequent “gap,” difficult to fit in otherwise busy curricula.

Although a number of “packages” including electrodes, data acquisition, and signal processing software provide global solutions attempting to overcome most of the usual hurdles, they require training for correct usage and interpretation of the generated outcomes. “Noise-believed-EMGs” and aberrant spectra, which can be attributed to any of the aforementioned issues, are commonly observed in experimental results obtained by novices. These observations underline the necessity for a sufficient understanding of how to overcome the inherent limitations of EMG systems. They remain primarily designed for experts, may not cover all EMG applications, and, like all software, suffer from the usual weaknesses of interfaces and user guides.

In sum, times to teach and learn and training-based skills pose limitations to the utilization of EMG as a clinical tool aimed at the prevention and reduction of work-related musculoskeletal disorders. Furthermore, unlike other signals such as the EKG or EEG, which present similar limitations but are extensively used as clinical tools, the EMG does not provide information about life-threatening conditions, although it can provide useful information about health- or profit-threatening conditions. Hence, despite some utilization for the investigation of MSDs as a research tool, the absence of immediate life-saving status has probably relegated the EMG to a secondary role in the clinical arsenal and is not frequently used by occupational therapists (private conversations).

**Figure 2:**
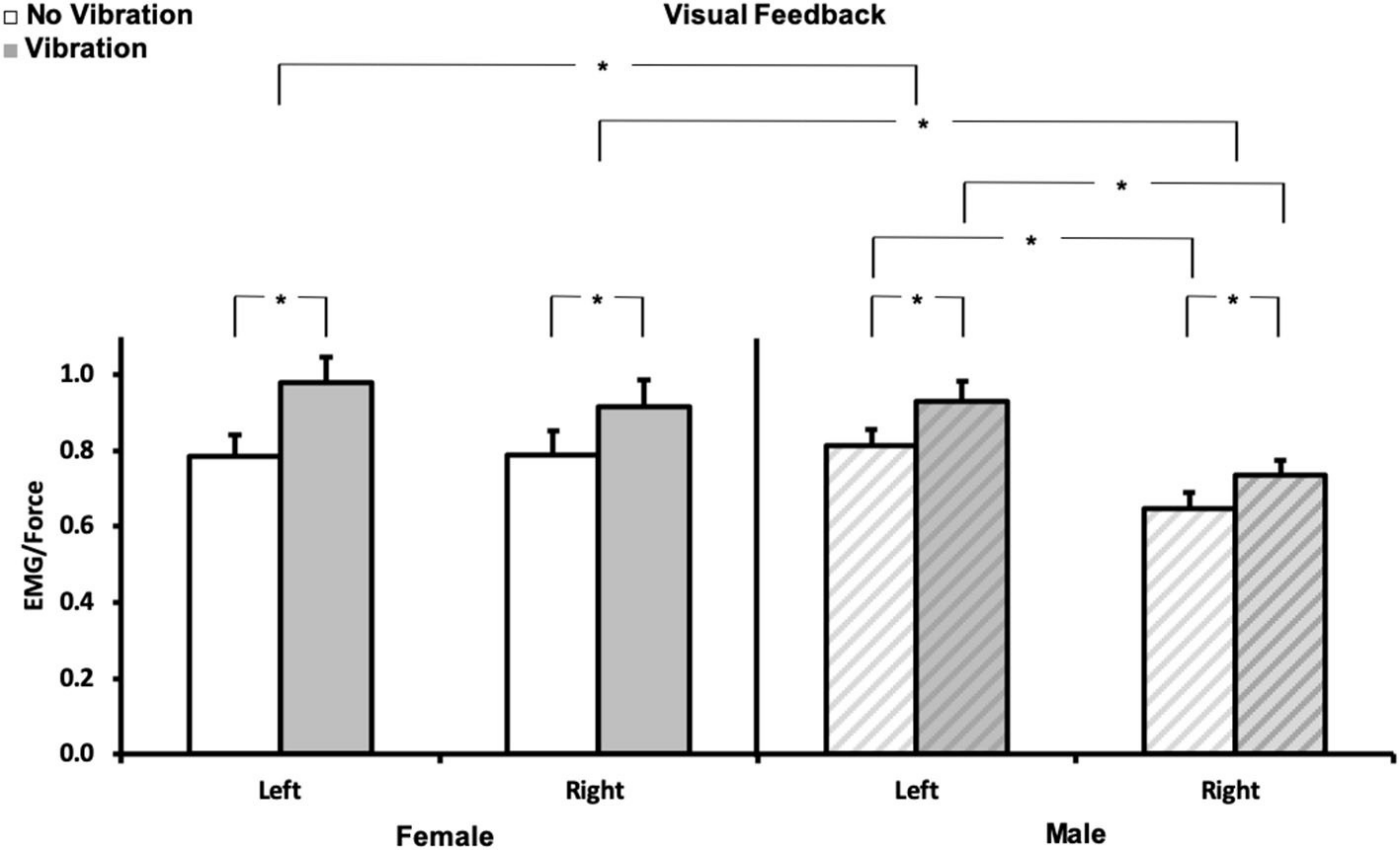
Normalized EMG/force. Mean (± SE) for each hand (left and right) for no vibration (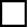) and vibration (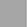) for females (*N* = 10, left panel), and males (*N* = 10, right panel 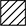). *p < 0.05. Each variable (EMG, force) was normalized to its corresponding 100% MVC. The required force exertion was 20% MVC.

To illustrate some of the issues mentioned above and the work necessary to broadly support clinical applications, one component of a recent EMG-based study is presented as an original example. This investigation required the one-to-one training of a doctoral student in ergonomics from electrode placement to signal processing and interpretation due to the discovery of EMG practice. Major attention focused on precise electrode placement, signal validation, filtering techniques, and mechanisms underlying EMG variations in context. The hypothesis was that asymmetry in the functioning of the upper limb sensorimotor system (24–27) stems, to some extent, from the difference in sensitivity of the proprioceptive feedback loop between the dominant and non-dominant arm. This difference in sensitivity can be expressed by the difference in gain of the respective sensory-motor loops (28). It is posited that alteration of this imbalance by age, stroke, injuries, or diseases can be used to identify further sensory and motor deficits and then propose individualized rehabilitation procedures. To validate our hypothesis, EMG activity of the left and right flexor digitorum superficialis (FDS) was recorded in dextral young adults during a static, visually controlled, grasp force task [see (25, 27) for procedure] maintained before and during the application of a 60-Hz vibration to the distal tendons of the wrist flexor muscles. Vibration elicits a response of muscle and cutaneous receptors [e.g., (29, 30)], respectively mediated by Ia and cutaneous pathways. This sensory feedback drives primarily, but not necessarily, the motoneurons homonymous to the source activated (31, 32) and modifies perception (33). As a result, the interplay of the recruited muscles (agonist/antagonists) requires an adjustment of the motor command to maintain the grip force constant (33, 34). On this basis, changes in the EMG/force ratio was considered to reflect changes in the “gain of the sensorimotor system” tested. The aim was to quantify the extent to which the EMG reflects the expected differences between the dominant and non-dominant hand. All tests were performed in a standardized seated upright posture. Visual feedback of the grip force (20% MVC) was presented on a vertical scale displayed on a computer screen. Force and EMG signals were normalized to each hand 100% MVC obtained before the experiment. Two practice trials preceded the three test trials for each hand. The EMG/Force ratio was used as the dependent variable. While on average grip force level did not vary much due to visual control, for both genders, the EMG/Force ratio was greater with than without vibration for both hands (*p* < 0.01). *Post-hoc* comparisons indicated that the EMG/force was greater for females than males for the right and left hand (*p* < 0.05) and for the left than the right hand for males (*p* < 0.002). The EMG/force was not significantly different (*p* > 0.05) between hands for females. These results are illustrated in Figure 2. In brief, they illustrate the sensorimotor asymmetries between the right dominant and left non-dominant hand in males and confirm a gender difference with much less asymmetry in females despite their higher sensitivity (greater sensorimotor gain), which validates our previous hypothesis concerning a gender-dependent difference in the gains of the left and right sensorimotor systems (24–28, 35). Despite the value of such results, their exploitation time, as a diagnostic or rehabilitation assessment tool, is not negligible. Indeed, with a well-trained “tester” the duration of the test itself and following data processing is usually no <1.5 h.

## Conclusion

A noise-like complexion makes the EMG, like other physiological signals, prone to confusion. Nevertheless, as evidenced by EMG quantification in the example provided above, the non-uniform gain of the sensorimotor proprioceptive systems of the upper limbs provides a number of insights concerning the functioning of sensory-motor loops and their contribution to force control. These include accessibility of homonymous motoneurons by Ia afferents, central control of sensory information, the gain of sensorimotor systems, and gender proprioceptive sensitivity. Each of these phenomena can be revealed by sEMGs and may be used to diagnose and monitor sensory and motor impairments resulting from aging, stroke, or other disease and injuries. To this end, the restoration of the disrupted “natural” balance/imbalance may be achieved via personalized rehabilitation procedures that consider the identified deficits. In Ergonomics, the methodology can be used to assess deficits resulting from work-related musculoskeletal disorders. Another application is the assessment of the muscle load induced by the operation of vibrating tools and thus tool selection. However, as indicated in section sEMG challenges and illustrative examples, the application of sEMG requires knowledge (e.g., EMG theory, signal processing, and neurophysiology), experience (e.g., electrode placement and signal morphology), and time (e.g., experiment design and procedure). These essential components may be deepened in doctoral studies but are not provided in clinical curricula such as physiotherapy and occupational therapy. Hence, broadening or amending the educational programs of physiotherapists, occupational therapists, movement scientists, and ergonomists (as future clinical users of sEMG) should prove useful. A lab course-specific training, including theory and hands-on practice, could be implemented. This could help resolve the recurrent comments from colleagues and professionals “We train them today, but they will work for the next 30 years and should be able to manage the huge technological changes that will take place… they should be able to read and understand papers and books in the field… we were not taught that in school, so it may not be too important… it was only an overview…” Although without a need to master the physics and mathematics of EMG, familiarization with basic concepts and technology should enable practitioners to translate scientific advances into clinical practice. However, if specialization is possible in some schools, getting a sufficient number of students in a specific subject remains a major issue in engineering and even biomedical engineering. Recruiting students from different schools or disciplines is already attempted but not very successful due to program requirements in terms of hours and content distribution and specific needs for research studies at the master or doctoral level. Hence, a new program focusing on clinical applications (including more extensively EMG as one of the tools) could be thought of in the realm of “health care” as is the case at the Delft University of Technology (DTU) in the Netherlands (see website). However, differences in the missions of Technical Universities in Europe (application oriented) and universities or Technology Institutes (generally labeled “school Name tech/IT”) in the USA (research oriented), which are beyond the scope of this paper, may add difficulties to the adaptation of such a model. For example, the “Health Care” program “CHEPS” at the University of Michigan is focused on engineering health care (management/operation/patient safety). Thus, implementation of the DTU model may be more easily achieved in schools dedicated to physio- or occupational-therapy and perhaps in Kinesiology, with adapted teaching of technical skills. Furthermore, it appears that robotics rehabilitation is a rapidly evolving trend in the USA and most likely other countries. Hence, the expansion of other clinical application tools such as the EMG may be currently constrained within universities. These comments reflect only observations related to colleagues' work.

Furthermore, health insurance systems usually assume that one short expedited measure fits all, which prevents refinement of our understanding of sensory or motor deficits and thus precludes utilization of the tool presented. For example, stroke-induced deficits are estimated by crude physical assessments, and clinical rehabilitation procedures are mostly the same for everybody despite a common failure to promote significant recovery (36–38). The sEMG would supply information for better assessment of deficits as well as rehabilitation progress and/or efficacy. Finally, the instrumentation necessary to conduct such an analysis is usually expensive due to limited demand and technically complex with scarce support.

## Data Availability Statement

The raw data supporting the conclusions of this article will be made available by the authors, without undue reservation.

## Ethics Statement

The studies involving human participants were reviewed and approved by University of Michigan Institutional Review Board. The participants provided their written informed consent to participate in this study.

## Author Contributions

All authors listed have made a substantial, direct and intellectual contribution to the work, and approved it for publication.

## Conflict of Interest

The authors declare that the research was conducted in the absence of any commercial or financial relationships that could be construed as a potential conflict of interest.
